# Disentangling the gut microbiome and inflammation in inflammatory bowel diseases: longitudinal observations from the IBSEN III study

**DOI:** 10.1093/ibd/izag051

**Published:** 2026-04-09

**Authors:** Maria G Maseng, Simen H Hansen, Olle Grännö, Corinna Bang, Charlotte Lund, Gert Huppertz-Hauss, Gøri Perminow, Jørgen Valeur, May-Bente Bengtson, Randi Opheim, Raziye Boyar, Svein O Frigstad, Tone Bergene Aabrekk, Trond Espen Detlie, Vendel A Kristensen, Vibeke Strande, Øistein Hovde, Øyvind Asak, Andre Franke, Jonas Halfvarsson, Marte L Høivik, Johannes R Hov

**Affiliations:** Institute of Clinical Medicine, Faculty of Medicine, University of Oslo, 0371 Oslo, Norway; Department of Gastroenterology, Division of Medicine, Oslo University Hospital, 0450 Oslo, Norway; Bio-Me AS, 0349 Oslo, Norway; Institute of Clinical Medicine, Faculty of Medicine, University of Oslo, 0371 Oslo, Norway; Department of Transplantation Medicine, Division of Surgery, Inflammatory Diseases and Transplantation, Norwegian PSC Research Centre, Oslo University Hospital, 0372 Oslo, Norway; Division of Surgery, Inflammatory Diseases and Transplantation, Research Institute of Internal Medicine, Oslo University Hospital, 0450 Oslo, Norway; Department of Laboratory Medicine, Clinical Microbiology, Faculty of Medicine and Health, Örebro University, Örebro, 701 82, Sweden; Institute of Clinical Molecular Biology, Christian-Albrechts-University of Kiel, Kiel, 24118 Kiel, Germany; Institute of Clinical Medicine, Faculty of Medicine, University of Oslo, 0371 Oslo, Norway; Department of Gastroenterology, Division of Medicine, Oslo University Hospital, 0450 Oslo, Norway; Department of Gastroenterology, Telemark Hospital Trust, Skien, 3710 Skien, Norway; Department of Pediatrics, Oslo University Hospital, Oslo, 0450 Oslo, Norway; Institute of Clinical Medicine, Faculty of Medicine, University of Oslo, 0371 Oslo, Norway; Unger-Vetlesen Institute, Lovisenberg Diaconal Hospital, 0456 Oslo, Norway; Department of Gastroenterology, Vestfold Hospital Trust, 3103 Tønsberg, Norway; Department of Gastroenterology, Division of Medicine, Oslo University Hospital, 0450 Oslo, Norway; Department of Public Health, Institute of Health and Society, University of Oslo, 0371 Oslo, Norway; Department of Medicine, Diakonhjemmet Hospital, 0370 Oslo, Norway; Department of Medicine, Bærum Hospital, Vestre Viken Hospital Trust, 1346 Gjettum, Norway; Institute of Clinical Medicine, Faculty of Medicine, University of Oslo, 0371 Oslo, Norway; Department of Gastroenterology, Tønsberg Hospital, Vestfold Hospital Trust, 3103 Tønsberg, Norway; Institute of Clinical Medicine, Faculty of Medicine, University of Oslo, 0371 Oslo, Norway; Department of Gastroenterology, Akershus University Hospital, 1474 Lørenskog, Norway; Institute of Clinical Medicine, Faculty of Medicine, University of Oslo, 0371 Oslo, Norway; Department of Gastroenterology, Division of Medicine, Oslo University Hospital, 0450 Oslo, Norway; Institute of Clinical Medicine, Faculty of Medicine, University of Oslo, 0371 Oslo, Norway; Unger-Vetlesen Institute, Lovisenberg Diaconal Hospital, 0456 Oslo, Norway; Department of Gastroenterology, Lovisenberg Diaconal Hospital, 0456 Oslo, Norway; Institute of Clinical Medicine, Faculty of Medicine, University of Oslo, 0371 Oslo, Norway; Department of Internal Medicine, Gjøvik Hospital, Innlandet Hospital Trust, 2380 Brumunddal, Norway; Department of Medicine, Lillehammer Hospital, Innlandet Hospital Trust, 2609 Lillehammer, Norway; Institute of Clinical Molecular Biology, Christian-Albrechts-University of Kiel, Kiel, 24118 Kiel, Germany; Department of Gastroenterology, Faculty of Medicine and Health, Örebro University, 70182 Örebro, Sweden; Institute of Clinical Medicine, Faculty of Medicine, University of Oslo, 0371 Oslo, Norway; Department of Gastroenterology, Division of Medicine, Oslo University Hospital, 0450 Oslo, Norway; Institute of Clinical Medicine, Faculty of Medicine, University of Oslo, 0371 Oslo, Norway; Department of Transplantation Medicine, Division of Surgery, Inflammatory Diseases and Transplantation, Norwegian PSC Research Centre, Oslo University Hospital, 0372 Oslo, Norway; Division of Surgery, Inflammatory Diseases and Transplantation, Research Institute of Internal Medicine, Oslo University Hospital, 0450 Oslo, Norway; Section of Gastroenterology, Department of Transplantation Medicine, Division of Surgery, Inflammatory Diseases and Transplantation, Oslo University Hospital, 0372 Oslo, Norway

**Keywords:** microbiome, IBD, longitudinal, calprotectin, symptoms

## Abstract

**Background and Aim:**

Despite the well-established involvement of the gut microbiome in inflammatory bowel disease (IBD), less is known about how the gut microbiome changes over time and how it varies with clinical disease activity and fecal calprotectin (f-calprotectin). To address this gap, we utilized samples from the population-based inception cohort of the Inflammatory Bowel Disease in South-Eastern Norway III (IBSEN III) study.

**Methods:**

Data and stool samples from study participants with IBD and symptomatic controls were collected at diagnosis and after 3, 6, and 12 months. Microbiome profiling of stool samples was performed targeting the V3-V4 region of the 16S rRNA gene, and a consensus-based approach of mixed models was employed for the longitudinal microbiome analysis.

**Results:**

We included 1251 samples from 744 patients with ulcerative colitis, 618 samples from 356 patients with Crohn’ s disease and 266 samples from 164 symptomatic non-IBD controls. In the IBD population, we observed that levels of f-calprotectin decreased over time, as did the patient-reported disease activity (*P* < .001). Distinct changes in the gut microbiome of IBD patients were observed throughout the first year, such as increased alpha diversity (*P* < .001) and significant taxonomic changes.

Notably, there was no covariation between the changes in alpha diversity and f-calprotectin or symptom score.

**Conclusion:**

The gut microbiome during the first year after IBD diagnosis showed changes that paralleled inflammation and clinical disease activity, albeit without covariation, suggesting that there may be a disease-driving impact of gut microbiome independent of inflammation and inflammation-driven symptoms.

Key Messages **What is already known?** 

The gut microbiome of individuals with IBD shows greater temporal variability compared to that of healthy individuals.

**What is new here?** 

During the first year after diagnosis, the IBD gut microbiome shifts toward increased abundance of beneficial bacterial taxa. However, reductions in fecal calprotectin and clinical symptoms do not coincide with increased microbial diversity, indicating partly independent processes.

**How can this study help patient care?** 

By demonstrating that gut microbiome alterations can occur independently of inflammation, this study identifies the microbiome as a potential therapeutic and monitoring target, paving the way for earlier and more individualized treatment strategies in IBD care.

## Introduction

Inflammatory bowel disease (IBD) is a chronic, relapsing inflammatory disorder of the gastrointestinal tract, affecting 0.5%–-1% of the global population.^1^ The disease is suggested to be initiated by microbial or environmental factors that trigger gut inflammation in genetically susceptible individuals, causing chronic intestinal inflammation.^2^ Extensive clinical and experimental research has implicated the gut microbiome in IBD pathogenesis.^3^ Both major subtypes of IBD, Crohns disease (CD) and ulcerative colitis (UC), are characterized by reduced microbial diversity, distinct alterations, and higher variability in microbiome composition compared to healthy individuals.^3^^,^^4^ Moreover, an IBD-like microbiome has been identified in individuals at high risk of developing the disease,^5–7^ supporting the notion that dysbiosis may precede and contribute to onset of symptoms, independent of overt inflammation. Differences in the IBD gut microbiome depending on disease activity state have been reported, illustrating the dynamic interplay between inflammation and microbial imbalance .^8^^,^^9^ On the other hand, intestinal inflammation increases oxygen levels in the gut lumen and thereby alters the local environment to favor growth of facultative anaerobes.^10^^,^^11^ Therefore, inflammation will push microbiome composition further toward a dysbiosis state, highlighting the difficulties in answering the question of cause or effect in studies of the IBD microbiome.

Despite extensive characterization of the IBD microbiome by subdiagnosis and disease status, the majority of studies are cross-sectional,^12^ and it is difficult to interpret the potential causal relationship between disease activity and the microbiome. This situation could possibly be improved by studying the relationship between the gut microbiome and disease activity over time. Endoscopy is the gold standard for monitoring IBD. However, fecal calprotectin (f-calprotectin), a maker of mucosal inflammation, is widely used as a less invasive option relevant for repeated measurements.^13^ Although f-calprotectin is nonspecific and exhibits a high degree of intra-individual variation and variation between different measurement methods, levels below 100 μg/g indicate mucosal remission, while levels above 250 μg/g suggest significant inflammation.^14^ Symptoms are also potential measurements of disease activity. The correlation between mucosal inflammation and patient-reported symptoms is typically poor in CD, though better in UC.^15^ For quantifying symptoms, patient-reported outcomes (PROs) are becoming the standard of measure. For CD, the recommended PRO2 measure is the sum of the weighted daily stool frequency (SF) and abdominal pain, whereas for UC it is SF and rectal bleeding.^15^

In this study, we used the population-based inception cohort IBSEN III with treatment-naive and newly diagnosed patients with IBD to study changes in markers of inflammation, symptoms, and gut microbiome during the first year after diagnosis. The aim was to study the co-correlation between disease activity and the microbiome to understand better whether these variables are independent factors or if gut microbiome alterations are driven by intestinal inflammation.

## Methods

### Study population, clinical definitions, and biochemical markers

The Inflammatory Bowel Disease in South-Eastern Norway (IBSEN) III study is a population-based observational inception cohort study that recruited participants suspected of having IBD. Participants were included during 2017-2019 at hospitals belonging to the South-Eastern health region in Norway, as detailed by Kristensen et al.^16^ All participants underwent standardized examinations according to study standard operating procedures (SOPs) at baseline and 1-year follow-up, including endoscopy, clinical assessments, and collection of biological samples. At 3 and 6 months there were no scheduled study visits at the hospitals, but participants responded to electronic questionnaires and participants from the largest centers were asked to provide fecal samples for microbiome analysis by mail. This study population comprised all adult (aged ≥18 years at time of diagnosis) participants in the IBSEN III cohort classified as having IBD or symptomatic controls who donated at least 1fecal sample.

### Fecal sample collection

Fecal samples were collected at home using a stool collection tube with DNA stabilizer (Invitek Diagnostics) for microbiome analysis and 1 dry tube for f-calprotectin analysis. Samples were returned to the hospital by mail and stored at −80 °C. A subset of the participants received a form for declaring the Bristol Stool Scale score of their fecal sample.

### Fecal sample processing and sequencing

Fecal samples were processed as described previously.^17^ In short, DNA was extracted in 2 batches using the QIAcube system. Protocol published by Fadrosh et al.^18^ was used for amplification of the V3-V4 region when preparing for 16S rRNA sequencing. The normalized and quality-controlled amplicons were pooled in 12 libraries and sequenced on the Illumina MiSeq platform using the v3 kit (Illumina) at the Norwegian Sequencing Centre in Oslo. Samples were kept randomized to minimize batch effects, although most baseline samples were processed in the first batch of extraction and sequencing libraries. Samples yielding less than 10 000 reads were resequenced.

### Bioinformatics processing

The full bioinformatics pipeline has been described previously.^17^ In short, the sequences were filtered, demultiplexed, trimmed, and merged using bbduk, cutadapt, and bbmerge.^19–21^ Denoising was conducted as implemented in QIIME2 2021.4 by Deblur^22^^,^^23^ and classification was performed using SILVA 138.^24–26^ Contaminants were removed and the remaining sequences were collapsed at the genus level before the output was exported from QIIME2 for subsequent analysis.

### Classification and definitions

The participants completed a symptom questionnaire that included questions regarding number of liquid stools, blood in stool (a factor, with 4 levels), defecation urgency, and stomach pain during (a factor, with 4 levels) the last 24 hours. The original PRO2 score relies on the number of stools,^15^ not liquid stools; thus, we calculated a modified PRO2 (mPRO2). For UC the score was composed by the sum of blood in stool and number of liquid stools, and for CD the score was composed of the sum of experienced stomach pain and number of watery stools. For f-calprotectin classification we used the cutoff at 250 µg/g to stratify between remission and ongoing disease activity.^27^

At the 1-year follow-up, we classified the participants with IBD into those having a severe or indolent disease course on the basis of a previously described composite endpoint composed of difficulties in controlling the inflammation and the development of IBD-related complications.^17^^,^^28^^,^^29^

IBD treatment data were collected at baseline and 1-year follow-up. Medication start date was recorded for all IBD treatments except for courses of steroids and budesonide. IBD medications were categorized into different groups as previously described,^30^^,^^31^ except for the medication 5-aminosalicylic acid (5-ASA) for which local and oral treatments were combined due to the expected effect on the microbiome.^32^

### Statistics

Data were handled within the safe environment for sensitive data (*Services for Sensitive Data)* at the University of Oslo. Linear mixed models were applied to account for repeated measures and confounders, with age, sex, and baseline antibiotic use as fixed effects and patient ID as a random effect, implemented in R (lme4 v1.1-35.3; lmerTest v3.1-3). To investigate longitudinal changes and time-dependent associations, time-lagged mixed models were used for handling repeated samples and confounders as described above. The intraindividual (alpha) diversity was measured by the Shannon diversity index, which was calculated on the rarefied dataset (10 003 reads per sample) using the phyloseq package in R (version 1.48.0). Differences in alpha diversity were assessed by linear- and time-lagged mixed models. Compositionality and stability (beta diversity) of samples cross-sectionally and over time were examined using the Bray-Curtis dissimilarity with the vegan (version 2.6-6.1), SEtools (version 1.12.0), and miaTime (version 0.99.8) packages. Stability in the gut microbiome over time was assessed by changes in beta diversity from baseline and from each previous time point. Differential abundance analysis was conducted using MaAsLin2 (version 1.18.0) and LinDA (version 0.2.0), both being mixed models that allow for handling repeated samples and adjustment for confounders. Only taxa that are differentially abundant with both methods are reported. The presented *q* values (*P* values adjusted for the false discovery rate) and model coefficients were taken from results obtained by using the LinDA program. Correlation analysis was conducted with the base R stats package, and Spearman’s rho was used for calculating correlation coefficients. Correlation refers to cross-sectional correlation, whereas covariation refers to the correlation between changes. Tables were made using gtsummary (version 1.7). Visualizations were made with ggplot2 (version 3.5.1) and combined by the package ggpubr (version 0.6.0). All analyses were conducted in RStudio using R version 4.2.3.

## Results

### Population

**Figure 1:**
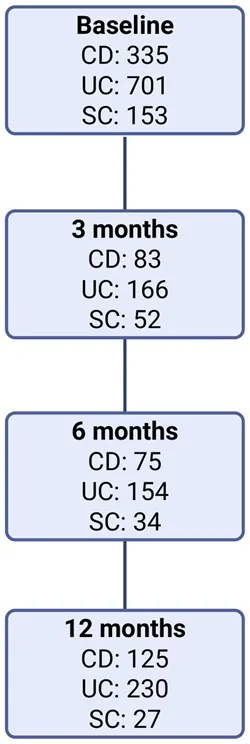
Flow chart of the number of participants who donated fecal samples at each time point. In total, the study population included 1264 individuals: 744 with UC, 356 with CD, and 164 symptomatic non-IBD controls.

The study population included 1264 participants: 744 with UC, 356 with CD, and 164 symptomatic non-IBD controls, each with at least 1 sample (Table 1). Among the participants diagnosed with UC, 204 provided samples at both baseline and 1 year, and 74 of these patients also donated samples at the 3- and 6-month follow-ups. Among participants with CD, 108 contributed samples at both baseline and 1 year, and 36 of these also provided samples at 3 and 6 months. Among the symptomatic non-IBD controls, 22 provided samples at both baseline and 1 year, while 18 provided samples at all 4 time points. The number of samples at each time point is shown in Figure 1. Compared to the overall adult IBSEN population, this study population was slightly older and had a higher proportion of females (Table S1).

**Table 1:** Characteristics of the study population.

Characteristic	**CD (n** = **356)**	**UC (n** = **744)**	**Symptomatic controls (n** = **164)**
**Age, median (IQR), y**	40 (28–53)	39 (28–52)	31 (24–40)
**Sex, *n* (%)**			
**Female**	208 (58)	356 (48)	82 (50)
**Male**	148 (42)	388 (52)	82 (50)
**Treatment-naive sample, *n* (%)**			
**0**	263 (74)	653 (88)	1 (0.6)
**1**	93 (26)	91 (12)	163 (99)
**Validated bacteria data at baseline and 12 m, *n* (%)**			
**0**	248 (70)	540 (73)	142 (87)
**1**	108 (30)	204 (27)	22 (13)
**Validated bacteria data at baseline, 3, 6, and 12 mo, *n* (%)**			
**0**	320 (90)	670 (90)	146 (89)
**1**	36 (10)	74 (9.9)	18 (11)
**Severe Disease Course, *n* (%)**			
**Severe course**	51 (16)	67 (9.6)	0 (0)
**Indolent course**	265 (84)	628 (90)	164 (100)
**Unknown**	40	49	0

Abbreviations: CD, Crohns disease; UC, ulcerative colitis.

### Microbiome during the first year

**Figure 2:**
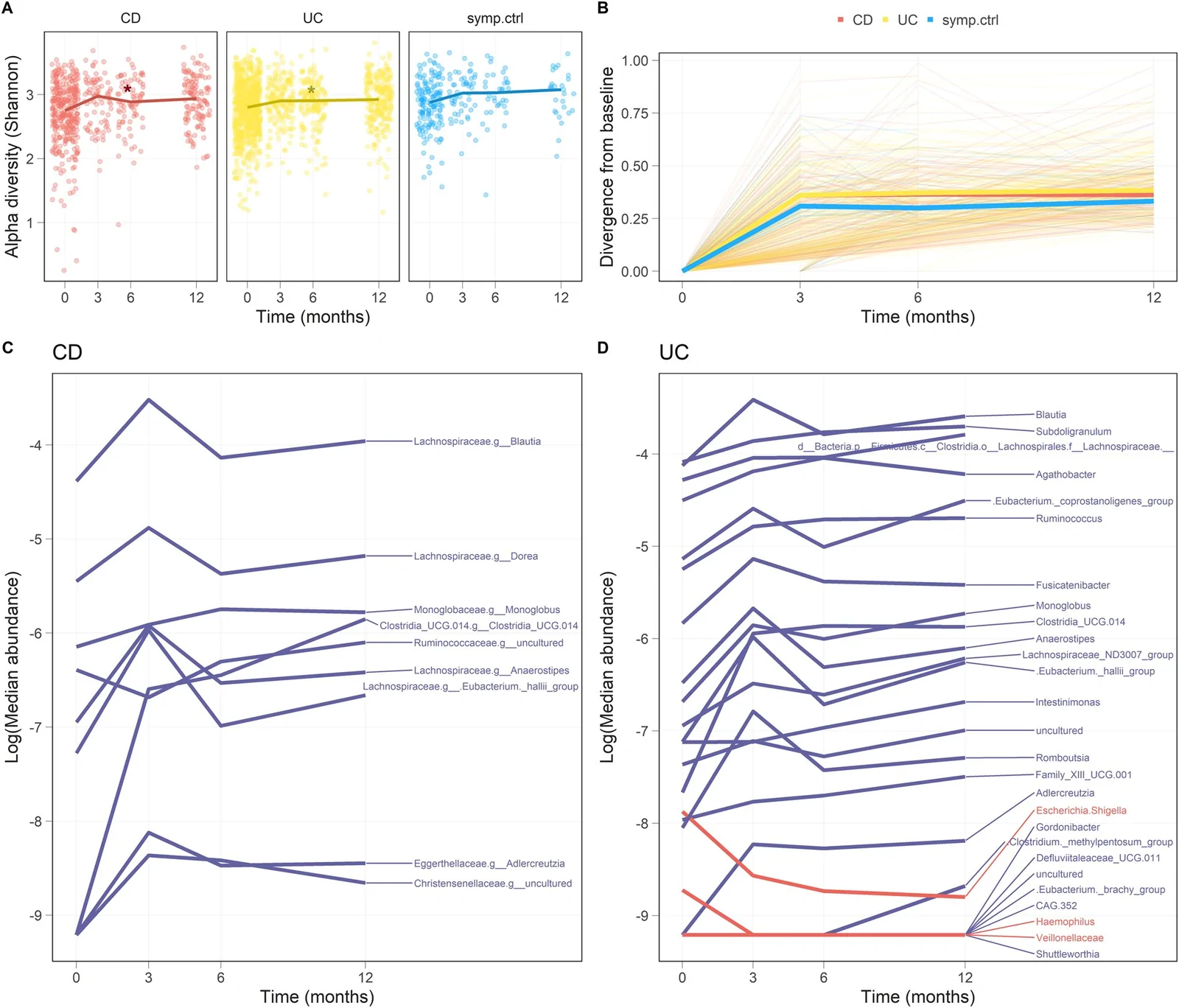
A. Alpha diversity (Shannon index) over time by diagnosis. Each dot represents a study participant; lines indicate the median per class. *denotes *P* < .05 from mixed-effects models. B. Bray-Curtis dissimilarity from baseline. Each line represents 1 patient; bold lines indicate the median per class. Variability was significantly higher in UC and CD compared with symptomatic non-IBD controls (adonis2, *P* < .05). C. Bacterial taxa with significant consensus-based differential abundance (MaAsLin2 and LinDA, *q* < 0.05) over time among patients with CD. Lines represent the median abundance per taxon, color coded by increasing or decreasing trend. D. Bacterial taxa with significant consensus-based differential abundance over time among patients with UC (MaAsLin2 and LinDA, *q* < 0.05). Lines represent the median abundance per taxon, color-coded by increasing or decreasing trend. CD, Crohns disease; UC, ulcerative colitis.

The intraindividual (alpha) diversity of the gut microbiome increased significantly over the first year in both CD (*P* = .001) and UC patients (*P* < .001, Figure 2A) but did not increase among the symptomatic controls. When assessing the global microbiome composition (beta diversity) and its variability over time, a significant difference in the degree of variability was observed between patients with UC and CD and the symptomatic controls, with the highest variability in UC and the lowest in symptomatic controls (Figure 2B). In differential abundance analysis, 9 taxa differed significantly over the first year in patients with CD (Figure 2C) and 27 taxa in those with UC (Figure 2D), while no taxa changed significantly in symptomatic controls.

### F-calprotectin levels and symptom severity

**Figure 3:**
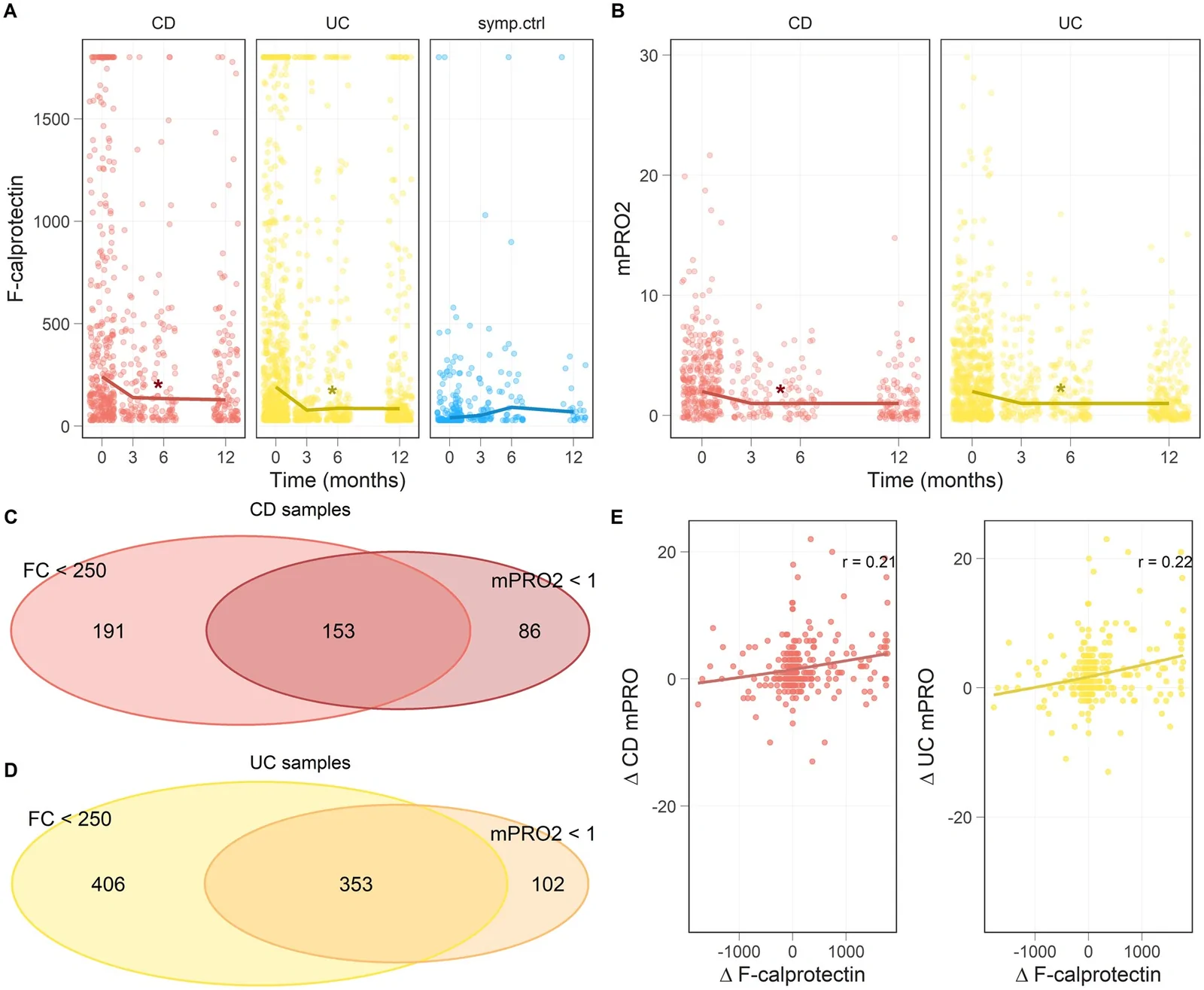
A. F-calprotectin over time by sub diagnosis. Each dot represents a participant; lines indicate the median per class. *Denotes *P* < .05 from mixed-effects models. B. mPRO2 scores over time by sub diagnosis. Same format as (A). C. Venn diagram of samples with f-calprotectin < 250 µg/g and/or mPRO2 < 1 among patients with CD. D. Venn diagram of samples with f-calprotectin < 250 µg/g and/or mPRO2 < 1 among patients with UC. E. Correlation between change in mPRO2 from baseline to 12 months and change in f-calprotectin in CD and UC (Spearman correlation; CD: *r* = 0.21, *P* = .0005; UC: *r* = 0.22, *P* = .0004). CD, Crohns disease; UC, ulcerative colitis.

The levels of F-calprotectin decreased throughout the first year in both CD and UC patients, while there was no change in samples from the symptomatic controls (Figure 3A). A Bristol stool score (BSS) was available for 48.6% of the samples at baseline and was weakly correlated with f-calprotectin (*r* = 0.13, *P* < .001) (Figure S1).

The majority of the included participants (88.7%) completed a questionnaire of symptoms at the time of stool sampling. Throughout the first year, there was a significant decline in the reported number of liquid stools, stomach pain, urgency to defecate, and reported blood in stool (Figure S2), and thereafter the mPRO2 score decreased significantly (*P* < .001, Figure 3B).

Considering all time points in a cross-sectional analysis, f-calprotectin and mPRO2 were moderately correlated (*r* = 0.33, *P* < .001, Figure S3), and among patients with UC a higher average f-calprotectin level was significantly associated with a higher average mPRO2 score, as evaluated by the time-lagged mixed model (*P* = .012). These increases were not observed in samples from CD patients (*P* = .51). Among all samples, 64% from CD and 59% from UC patients had an f-calprotectin below 250 µg/g and reported symptoms or reported no symptoms but had f-calprotectin above 250 µg/g (Figure 3C and D). There was a weak correlation (*r* = 0.21–0.22, *P* < .001) between the changes (deltas) in f-calprotectin and mPRO2 score from baseline to 1 year (Figure 3E), with similar values in the subset with baseline f-calprotectin above 250 µg/g (*r* = 0.19, *P* = .04 and *r* = 0.24, *P* = .01 for CD and UC, respectively, Figure S4). Through the time-lagged analysis, we observed that a reduction in f-calprotectin significantly predicted a lower mPRO2 score at the next visit (*P* = .011). When stratified by IBD subtype, this association was not significant among participants with CD (*P* = .445) but remained significant in UC (*P* = .011).

### Relationship between microbiome, f-calprotectin, and symptoms

**Figure 4:**
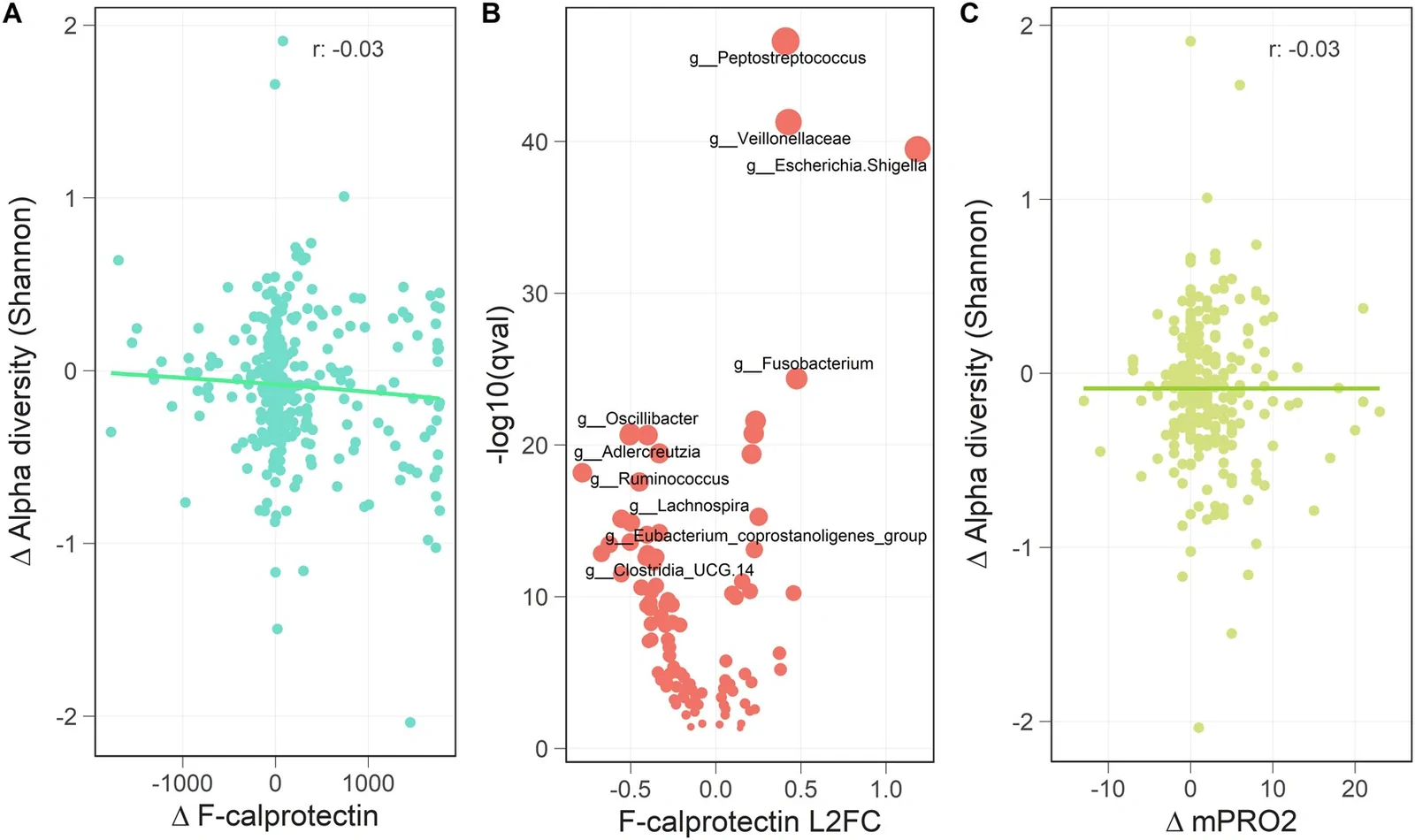
A. Correlation between change in alpha diversity (Shannon index) from baseline to 12 months and change in f-calprotectin. Spearman correlation: *r* = −0.03, *P* = .6. B. Bacterial taxa with significant consensus-based differential abundance by f-calprotectin, independent of time point (MaAsLin2 and LinDA, *q* < 0.05). The *x*-axis represents the log2 fold change and the *y*-axis the -log10 of the *q*-value. C. Correlation between change in alpha diversity (Shannon index) from baseline to 12 months and change in mPRO2. Spearman correlation: *r* = −0.04, *P* = .5.

In the time-lagged analysis, participants with overall higher microbiome diversity had lower fecal calprotectin levels (*P* = .022), but intra-individual (alpha) diversity only weakly correlated with levels of f-calprotectin (*r* = −0.14, *P* < .001, Figure S5A). There was no correlation between the observed change (delta) in alpha diversity and f-calprotectin over the first year (*r* = −0.03, *P* = .6, Figure 4A), and changes in alpha diversity did not predict change in levels of f-calprotectin (*P* = .52). The levels of f-calprotectin were highly stable over time and were best explained by its previous measurement (*P* < .001). Multiple bacterial taxa associated significantly with levels of f-calprotectin (105 taxa *q* < 0.05, Figure 4B and Table S2) when adjusting for repeated samples and confounders, with *Escherichia-Shigella* as the statistically strongest association.

Patients with higher overall diversity had lower symptom burden (*P* = .027), and a weak correlation pattern was found between alpha diversity and the symptom score mPRO2 at any given time point (*r* = −0.2, *P* < .001, Figure S^5^B). However, there was no correlation between the changes (deltas) in alpha diversity and mPRO2 the first year (*r* = −0.04, *P* = .5, Figure 4C). Furthermore, a change in alpha diversity did not predict change in mPRO2 score (*P* = .64), and the symptom scoring was significantly persistent over time (*P* < .001). Multiple taxa were differentially abundant based on the mPRO2 score while adjusting for repeated samples and confounders (Table S3 and S4).

### Medications per se as a contributing factor to microbiome changes

The participants donated their fecal samples shortly after starting treatment. For CD patients the median was 17 days, whereas for UC patients the median was 9 days. To investigate the effect of medications, we focused only on individuals with a treatment-naive sample available and at least 1 paired sample at a later point. Given the variable treatment strategies the first year after the IBD diagnosis and limited numbers of follow-up samples, we looked specifically at the largest first-treatment group, UC patients treated with 5-ASA (Figure S6). Treatment-naive fecal samples were available from 77 patients before they started oral or local 5-ASA therapy. There was no difference in alpha diversity before vs after 5-ASA initiation (*P* = .80), but we observed global compositional differences (*P* = .030). Differential abundance analysis revealed 4 genera to differ significantly when we compared samples collected before and after 5-ASA therapy (increased *Blautia* and decreased *Akkermansia, Hydrogenoanaerobacterium, uncultured Firmicutes*).

### Increased gut microbiome variability in patients with severe disease course

**Figure 5:**
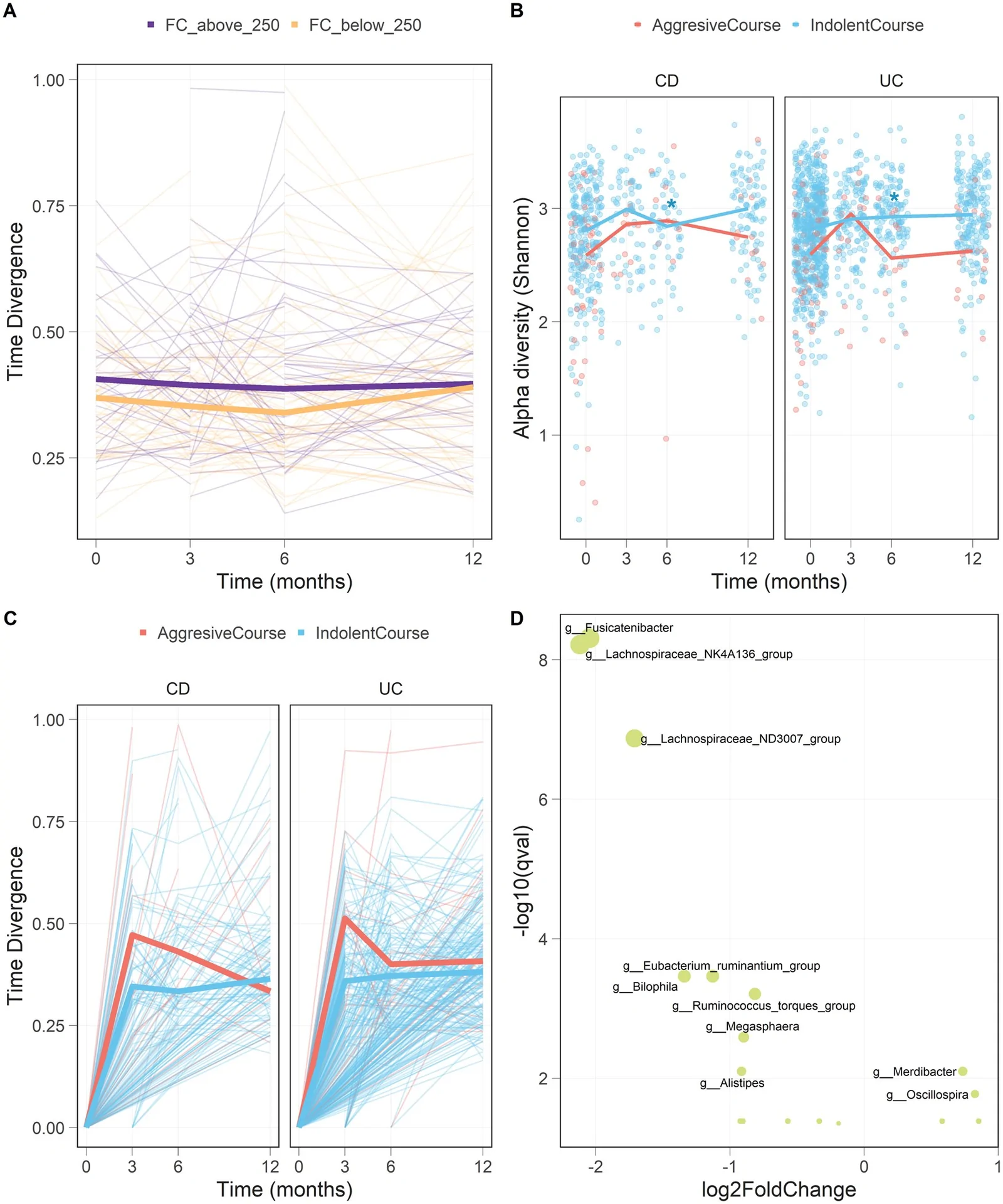
A. Bray-Curtis dissimilarity from the previous time point stratified by stable f-calprotectin above vs below 250 µg/g. Variability differed significantly (adonis2, *P* < .05). B. Alpha diversity (Shannon index) over time by subdiagnosis and disease course. Significant increases were observed in indolent CD and UC, but not in aggressive disease. C. Bray-Curtis dissimilarity from baseline by subdiagnosis and disease course. Variability differed significantly in UC (adonis2, *P* < .05). D. Consensus-based differentially abundant taxa distinguishing UC with low activity and symptomatic non-IBD controls. The *x*-axis represents the log2 fold change and *y*-axis the -log10 of the *q*-value. CD, Crohns disease; UC, ulcerative colitis.

We classified participants based on stable-high or stable-low levels of f-calprotectin and found that the stable-high group had significantly higher variability in microbiome composition over time when adjusting for diagnosis (*P* = .001, Figure 5A).

A severe disease course was observed in 17% of patients with CD and 9.5% among patients with UC (Table 1). Alpha diversity was generally lower among those having a severe disease course than those with an indolent course (*P* < .001), with an initial increase at 3 months but with a subsequent trend toward reduction (Figure 5B). Considering microbial stability by comparing the Bray-Curtis dissimilarity between each sample and its baseline, we found no significant difference in degree of variability over time among CD patients having an indolent or severe disease course (*P* = .11). In patients with UC the variability in composition over time was significantly higher in those with severe disease than in those with an indolent disease trajectory (*P* = .009, Figure 5C).

### Gut microbiome in patients with low f-calprotectin

To test whether microbiome changes were driven by inflammation, we adjusted the longitudinal differential abundance models for f-calprotectin by including it as a covariate. In CD patients, *Eubacterium hallii*, *Anaerostipes*, *Blautia*, and *Adlercreutzia* remained significantly altered over the first year. In UC patients, 19 of 27 taxa retained significance after adjustment (Figure S7).

Furthermore, we stratified our population to samples with f-calprotectin levels below 250 µg/g. The alpha diversity was reduced in both CD patients (*P* = .002) and UC patients (*P* = .011) compared to symptomatic controls, regardless of timepoint. Among taxa, only *Oscillospiraceae UCG-003* differed between the low-calprotectin CD patients and controls, whereas 17 taxa were differentially abundant in the UC patients (Figure 5D).

## Discussion

In the present study, we report overall increases in microbial diversity over the first year after IBD diagnosis that parallelled significant reductions in f-calprotectin and symptom scores. Multiple bacteria taxa increased over time, many of which were negatively associated with f-calprotectin and mPRO2. However, the correlations between microbiome diversity, f-calprotectin, and mPRO2 were only weak to moderate, and changes in microbiome composition did not explain changes in f-calprotectin or mPRO2. Initiation of therapy with 5-ASA was associated with unique microbiome changes that did not overlap with the overall changes in taxa over time. Furthermore, in the subgroup of UC patients with a severe disease course, we observed less stable microbiome composition over time compared to patients with an indolent disease course. Finally, in a comparison of IBD patients with low f-calprotectin and symptomatic controls, the diagnosis-specific signal remained in UC patients, while it almost disappeared in CD patients. Taken together, these findings indicate a distinct correlation between gut inflammation, symptoms, and microbiome composition. However, the correlations were not strong, and we detected no covariation, suggesting that the gut microbiome may have a disease-driving impact that is independent of inflammation and inflammation-driven symptoms.

The symptoms and f-calprotectin levels were reduced over time as the participants joined the study and started therapy. Especially for f-calprotectin, the largest reduction took place from diagnosis to 3 months, which is in line with data from other studies.^33^^,^^34^ This finding is also in line with the observation that the majority of the participants in the investigated IBSEN III cohort reported a disease course best described as highly active initially, followed by remission or mild disease.^30^^,^^31^

Patients with IBD had a more variable microbiome composition than healthy patients, and this finding is to a large degree influenced by location of disease, surgery, and medications.^4^ Over the first year of investigation of the newly diagnosed patients with IBD, we observed an increasing diversity over time, which could be interpreted as a development toward a “less IBD-like” microbiome. During the first 12 months we also observed more variability (measured by Bray-Curtis dissimilarity) in the IBD microbiome compared to symptomatic controls. Independent of diagnosis, participants with stable high f-calprotectin (above 250 µg/g) throughout the year had higher degree of intra-individual variation in their microbiome composition than those with low f-calprotectin. Through cross-sectional analysis it has been shown that metagenomic variation and lower alpha diversity correlates strongly to f-calprotectin,^35^ whereas longitudinal analyses revealed minimal effect on the microbiome dynamics of f-calprotectin.^4^ On the other hand, it was reported that the UC microbiome is highly stable within individuals across disease stages, activity levels, and treatment escalation both at diagnosis and in patients with established IBD, describing the gut microbiome composition with the Bray-Curtis dissimilarity index by characterizing gut microbiome composition.^34^ Another study, using unweighted UniFrac dissimilarity reported that people with UC in long term remission (defined by Mayo, SCCAI and histology) presents a microbiome profile similar to healthy controls.^36^ However, the within-patient fluctuations (measured by Bray-Curtis dissimilarity) were shown to differ by disease activity^9^ and to be larger in IBD than in healthy individuals.^4^^,^^9^ We found a significant change in the IBD gut bacterial composition over time, primarily characterized by an increase in bacteria considered beneficial for gut health. In contrast, a paediatric cohort study (IBD *n* = 104) that investigated the microbiome from treatment-naive patients to 6 weeks post-treatment reported no significant increase in diversity, though they did observe a decrease in the abundance of *Escherichia coli* and Proteobacteria among other taxonomic changes.^37^ In UC, we observed a decrease in the facultatively anaerobic genera *Escherichia-Shigella* and *Haemophilus* (both within the *Gammaproteobacteria*). These organisms can thrive in both oxygen-rich and -poor environments and often outcompete obligate anaerobes. As demonstrated by Winter et al. nitrate produced during the host inflammatory response can be utilized by bacteria such as *E. coli*, giving them a competitive advantage over obligate anaerobes and explaining their enrichment during inflammation.^38^ Notably, the increasing taxa in our cohort were predominantly from the *Clostridia* class and the order *Coriobacteriales*, both of which are known to contribute to gut homeostasis and are typical members of a healthy microbiome.^39^^,^^40^ The longitudinal changes we observed thus suggest a shift toward a more homeostatic gut environment, and we show that there are changes in the microbiome independent of what can be explained by decrease in f-calprotectin.

Both the symptom score and f-calprotectin had a weak or moderate correlation to gut microbiome composition, and a higher diversity was associated with lower f-calprotectin and symptom burden. However, there was no covariation between symptoms and f-calprotectin and the gut microbiome during the first year. The cross-sectional associations between symptoms/f-calprotectin and gut microbiome are very much in line with previous studies on both symptoms^41^ and f-calprotectin,^35^ for which IBD with f-calprotectin above 250 µg/g is associated with reduced microbiome diversity compared to those with IBD and lower f-calprotectin.^9^ In CD, it has been reported that dysbiosis was associated with clinical score and symptoms (HBI), independent of mucosal inflammation (f-calprotectin).^41^ Others investigators who have used different definitions of active IBD, such as endoscopic score (Mayo), clinical score (CDAI/HBI), or symptom score (SCCAI), have shown that the gut microbiome in IBD with low f-calprotectin was less different from that in symptomatic controls and reported differences in the fecal microbiome by the different definitions of disease activity.^36^^,^^41–43^

Despite these cross-sectional correlations, changes in microbial diversity did not imply a change in f-calprotectin or change in symptom score by the longitudinal analysis. This finding is in line with those by Halfvarson et al. who reported that the degree of inflammation was did not explain the difference in microbiome composition between healthy participants and patients with IBD.^4^ Overall, these findings suggests that the processes related to symptoms, inflammation, and microbiome composition are in part independent. Importantly, it is well known that patient-reported symptoms do not necessarily correlate strongly with mucosal inflammation in IBD.^15^^,^^44^

Direct effects of drug therapy may represent another factor changing the gut microbiome.^45–47^ Among patients with IBD, medication has been shown to explain more variation in microbial composition than IBD subtype, and changes in medication regime may affect the volatility of the microbiome.^4^^,^^48^ The IBD treatment regimens are individualized and vary greatly, and our study design did not allow us to fully explore this interplay. However, we did observe microbiome-specific differences before and after 5-ASA initiation. The complete mechanistic effect of 5-ASA therapy is unknown, but the medication has been shown to alter the conditions in the intestine to a less oxygenic environment, hence improving the conditions for obligative anaerobic bacteria.^49^ We observed a decrease in *Akkermansia,* which is in line with studies investigating treated patients with UC.^12^^,^^36^ Eckenberg et al. reported that the health-associated bacteria were affected by medications to a larger extent than the disease-associated bacteria,^48^ which is in line with our findings.

This study longitudinally assessed gut microbiome shifts during the first year following an IBD diagnosis, leveraging the large cohort of newly diagnosed patients from the IBSEN III study. A major strength of this study is the availability of repeated fecal samples collected from a substantial proportion of these participants. Furthermore, we have used robust consensus-based differential abundance analysis tools, as recommended by Nearing et al.,^50^ while adjusting for confounders and repeated sampling. The stool samples for microbiome profiling and f-calprotectin quantification were collected simultaneously, whereas the patient-reported data may be from a different previous 24 hours than the stool samples, which may have affected the investigated relationship between the sources. Our study had several limitations. As our study population ranges from treatment-naive individuals with active disease to newly diagnosed patients undergoing treatment and those in stable remission, this adds complexity to our findings, while the use of a slightly modified PRO2 score limits comparisons to other studies. Another limitation is that we did not adjust for important environmental variables known to influence the microbiome composition, such as diet or medications, and the fraction of participants delivering fecal samples at 3 and 6 months was also low. Microbiome profiling was conducted on the taxonomic genus level, a limitation with the 16S rRNA amplicon sequencing. Furthermore, 16S rRNA amplicon sequencing does not allow for functional analyses. Future studies should investigate the longitudinal functional implications of microbiome changes within specific IBD phenotypes.

## Conclusions

In patients during the first year after an IBD diagnosis, we observed significant reductions in f-calprotectin levels and symptom burden in parallel with gut microbiome alterations. Although a higher alpha diversity was associated with lower levels of f-calprotectin and symptom burden, a change in diversity did not predict a change in the mPRO2 score or f-calprotectin, suggesting a disease-driving impact of the gut microbiome independent of inflammation and symptoms.

## Supplementary Material

izag051_Supplementary_Data

## Data Availability

Available upon reasonable request.

## References

[1] Hracs L , Windsor JW, Gorospe J, et al; Global IBD Visualization of Epidemiology Studies in the 21st Century (GIVES-21) Research Group. Global evolution of inflammatory bowel disease across epidemiologic stages. Nature. 2025;642:458-466. 10.1038/s41586-025-08940-0 40307548 PMC12158780

[2] Wang R , Li Z, Liu S, Zhang D. Global, regional and national burden of inflammatory bowel disease in 204 countries and territories from 1990 to 2019: a systematic analysis based on the Global Burden of Disease Study 2019. BMJ Open. 2023;13:e065186. 10.1136/bmjopen-2022-065186 PMC10069527 36977543

[3] Iliev ID , Ananthakrishnan AN, Guo CJ. Microbiota in inflammatory bowel disease: mechanisms of disease and therapeutic opportunities. Nat Rev Microbiol. 2025;23:509-524. 10.1038/s41579-025-01163-0 40065181 PMC12289240

[4] Halfvarson J , Brislawn CJ, Lamendella R, et al Dynamics of the human gut microbiome in inflammatory bowel disease. Nat Microbiol. 2017;2:17004. 10.1038/nmicrobiol.2017.4 28191884 PMC5319707

[5] Raygoza Garay JA , Turpin W, Lee S-H, et al; CCC GEM Project Research Consortium. Gut microbiome composition is associated with future onset of Crohn’s disease in healthy first-degree relatives. Gastroenterology. 2023;165:670-681. 10.1053/j.gastro.2023.05.032 37263307

[6] Brand EC , Klaassen MAY, Gacesa R, et al; Dutch TWIN-IBD consortium and the Dutch Initiative on Crohn and Colitis. Healthy cotwins share gut microbiome signatures with their inflammatory bowel disease twins and unrelated patients. Gastroenterology. 2021;160:1970-1985. 33476671 10.1053/j.gastro.2021.01.030

[7] Jacobs JP , Spencer EA, Helmus DS, et al Age-related patterns of microbial dysbiosis in multiplex inflammatory bowel disease families. Gut. 2024;73:1953-1964. 10.1136/gutjnl-2024-332475 39122361 PMC11560537

[8] Kang D-Y , Park J-L, Yeo M-K, et al Diagnosis of Crohn’s disease and ulcerative colitis using the microbiome. BMC Microbiol. 2023;23:336. 10.1186/s12866-023-03084-5 37951857 PMC10640746

[9] Clooney AG , Eckenberger J, Laserna-Mendieta E, et al Ranking microbiome variance in inflammatory bowel disease: a large longitudinal intercontinental study. Gut. 2021;70:499-510. 10.1136/gutjnl-2020-321106 32536605 PMC7873428

[10] Ni J , Wu GD, Albenberg L, Tomov VT. Gut microbiota and IBD: causation or correlation? Nat Rev Gastroenterol Hepatol. 2017;14:573-584. 10.1038/nrgastro.2017.88 28743984 PMC5880536

[11] Lee JY , Bays DJ, Savage HP, Bäumler AJ. The human gut microbiome in health and disease: time for a new chapter? Infect Immun. 2024;92:e0030224. 10.1128/iai.00302-24 39347570 PMC11556149

[12] Pittayanon R , Lau JT, Leontiadis GI, et al Differences in gut microbiota in patients with vs without inflammatory bowel diseases: a systematic review. Gastroenterology. 2020;158:930-946.e1. 10.1053/j.gastro.2019.11.294 31812509

[13] D’Amico F , Nancey S, Danese S, Peyrin-Biroulet L. A practical guide for faecal calprotectin measurement: myths and realities. J Crohns Colitis. 2021;15:152-161. 10.1093/ecco-jcc/jjaa093 32392336

[14] Bodelier AGL , Jonkers D, van den Heuvel T, et al High percentage of IBD patients with indefinite fecal calprotectin levels: additional value of a combination score. Dig Dis Sci. 2017;62:465-472. 10.1007/s10620-016-4397-6 27933473 PMC5258807

[15] Turner D , Ricciuto A, Lewis A, et al; International Organization for the Study of IBD. STRIDE-II: An update on the Selecting Therapeutic Targets in Inflammatory Bowel Disease (STRIDE) Initiative of the International Organization for the Study of IBD (IOIBD): determining therapeutic goals for treat-to-target strategies in IBD. Gastroenterology. 2021;160:1570-1583. 10.1053/j.gastro.2020.12.031 33359090

[16] Kristensen VA , Opheim R, Perminow G, et al Inflammatory bowel disease in South-Eastern Norway III (IBSEN III): a new population-based inception cohort study from South-Eastern Norway. Scand J Gastroenterol. 2021;56:899-905. 10.1080/00365521.2021.1922746 34154494

[17] Hansen SH , Maseng MG et al Fecal microbiome reflects disease state and prognosis in inflammatory bowel disease in an adult population-based inception cohort. Inflamm Bowel Dis. 2025. 10.1093/ibd/izaf060 PMC12491950 40285477

[18] Fadrosh DW , Ma B, Gajer P, et al An improved dual-indexing approach for multiplexed 16S rRNA gene sequencing on the Illumina MiSeq platform. Microbiome. 2014;2:6. 10.1186/2049-2618-2-6 24558975 PMC3940169

[19] Bushnell B, SourceForge [Internet]. 2025 [cited 2025 June 20]. BBMap; Available from: https://sourceforge.net/projects/bbmap/

[20] Martin M. Cutadapt removes adapter sequences from high-throughput sequencing reads. EMBnet j. 2011;17:10-12. 10.14806/ej.17.1.200

[21] Bushnell B , Rood J, Singer E. BBMerge - Accurate paired shotgun read merging via overlap. PLoS One. 2017;12:e0185056. 10.1371/journal.pone.0185056 29073143 PMC5657622

[22] Amir A , McDonald D, Navas-Molina JA, et al Deblur rapidly resolves single-nucleotide community sequence patterns. mSystems. 2017;2:e00191-16. 10.1128/mSystems.00191-16 28289731 PMC5340863

[23] Bolyen E , Rideout JR, Dillon MR, et al Reproducible, interactive, scalable and extensible microbiome data science using QIIME 2. Nat Biotechnol. 2019;37:852-857. 10.1038/s41587-019-0209-9 31341288 PMC7015180

[24] Quast C , Pruesse E, Yilmaz P, et al The SILVA ribosomal RNA gene database project: improved data processing and web-based tools. Nucleic Acids Res. 2013;41:D590-596. 10.1093/nar/gks1219 23193283 PMC3531112

[25] Bokulich NA , Kaehler BD, Rideout JR, et al Optimizing taxonomic classification of marker-gene amplicon sequences with QIIME 2’s q2-feature-classifier plugin. Microbiome. 2018;6:90. 10.1186/s40168-018-0470-z 29773078 PMC5956843

[26] Robeson MS , O’Rourke DR, Kaehler BD, et al RESCRIPt: Reproducible sequence taxonomy reference database management. PLoS Comput Biol. 2021;17:e1009581. 10.1371/journal.pcbi.1009581 34748542 PMC8601625

[27] Kristensen V , Røseth A, Ahmad T, Skar V, Moum B. Fecal calprotectin: a reliable predictor of mucosal healing after treatment for active ulcerative colitis. Gastroenterol Res Pract. 2017;2017:2098293. 10.1155/2017/2098293 29225617 PMC5684574

[28] Strande V , Hagen M, Lund C, et al Predicting severe inflammatory bowel disease: a risk matrix model based on the IBSEN III inception cohort. Scand J Gastroenterol. 2025;60:716-727. 40483585 10.1080/00365521.2025.2512591

[29] Ling Lundström M , Peterson C, Hedin CRH, et al; BIOIBD Consortium. Faecal biomarkers for diagnosis and prediction of disease course in treatment-naïve patients with IBD. Aliment Pharmacol Ther. 2024;60:765-777. 10.1111/apt.18154 38997818

[30] Strande V , Lund C, Hagen M, et al Clinical course of ulcerative colitis: Frequent use of biologics and low colectomy rate first year after diagnosis-results from the IBSEN III inception cohort. Aliment Pharmacol Ther. 2024;60:357-368. 10.1111/apt.18097 38837289

[31] Lund C , Strande V, Hagen M, et al Low surgery rates in early crohn’s disease: results from a prospective population-based inception cohort-the inflammatory bowel disease in south-eastern Norway III study. Inflamm Bowel Dis. 2024. 10.1093/ibd/izae297 PMC12342730 39699202

[32] Dahl J-U , Gray MJ, Bazopoulou D, et al The anti-inflammatory drug mesalamine targets bacterial polyphosphate accumulation. Nat Microbiol. 2017;2:16267. 10.1038/nmicrobiol.2016.267 28112760 PMC5514548

[33] Constantine-Cooke N , Monterrubio-Gómez K, Plevris N, et al Longitudinal fecal calprotectin profiles characterize disease course heterogeneity in Crohn’s disease. Clin Gastroenterol Hepatol. 2023;21:2918-2927.e6. 10.1016/j.cgh.2023.03.026 37004971

[34] Öhman L , Lasson A, Strömbeck A, et al Fecal microbiota dynamics during disease activity and remission in newly diagnosed and established ulcerative colitis. Sci Rep. 2021;11:8641. 10.1038/s41598-021-87973-7 33883600 PMC8060394

[35] Franzosa EA , Sirota-Madi A, Avila-Pacheco J, et al Gut microbiome structure and metabolic activity in inflammatory bowel disease. Nat Microbiol. 2019;4:293-305. 10.1038/s41564-018-0306-4 30531976 PMC6342642

[36] Herrera-deGuise C , Varela E, Sarrabayrouse G, et al Gut microbiota composition in long-remission ulcerative colitis is close to a healthy gut microbiota. Inflamm Bowel Dis. 2023;29:1362-1369. 10.1093/ibd/izad058 37655859

[37] de Meij TGJ , de Groot EFJ, Peeters CFW, et al Variability of core microbiota in newly diagnosed treatment-naïve paediatric inflammatory bowel disease patients. PLoS One. 2018;13:e0197649. 10.1371/journal.pone.0197649 30102706 PMC6089417

[38] Winter SE , Winter MG, Xavier MN, et al Host-derived nitrate boosts growth of E. coli in the inflamed gut. Science. 2013;339:708-711. 10.1126/science.1232467 23393266 PMC4004111

[39] Lee JY , Tsolis RM, Bäumler AJ. The microbiome and gut homeostasis. Science. 2022;377:eabp9960. 10.1126/science.abp9960 35771903

[40] Loftus M , Hassouneh SAD, Yooseph S. Bacterial associations in the healthy human gut microbiome across populations. Sci Rep. 2021;11:2828. 10.1038/s41598-021-82449-0 33531651 PMC7854710

[41] Buffet-Bataillon S , Landreau C, Siproudhis L, Cattoir V, Bouguen G. Bacterial gut dysbiosis is associated with Crohn’s disease symptoms but not with elevated fecal calprotectin. Clin Res Hepatol Gastroenterol. 2021;45:101669. 10.1016/j.clinre.2021.101669 33744412

[42] Zheng J , Sun Q, Zhang M, et al Noninvasive, microbiome-based diagnosis of inflammatory bowel disease. Nat Med. 2024;30:3555-3567. 10.1038/s41591-024-03280-4 39367251 PMC11645270

[43] Buffet-Bataillon S , Durão G, Le Huërou-Luron I, et al Gut microbiota dysfunction in Crohn’s disease. Front Cell Infect Microbiol. 2025;15:1540352. 10.3389/fcimb.2025.1540352 40007605 PMC11850416

[44] Lloyd-Price J , Arze C, Ananthakrishnan AN, et al; IBDMDB Investigators. Multi-omics of the gut microbial ecosystem in inflammatory bowel diseases. Nature. 2019;569:655-662. 10.1038/s41586-019-1237-9 31142855 PMC6650278

[45] Ananthakrishnan AN , Luo C, Yajnik V, et al Gut microbiome function predicts response to anti-integrin biologic therapy in inflammatory bowel diseases. Cell Host Microbe. 2017;21:603-610.e3. 10.1016/j.chom.2017.04.010 28494241 PMC5705050

[46] Vich Vila A , Collij V, Sanna S, et al Impact of commonly used drugs on the composition and metabolic function of the gut microbiota. Nat Commun. 2020;11:362. 10.1038/s41467-019-14177-z 31953381 PMC6969170

[47] Jackson MA , Verdi S, Maxan M-E, et al Gut microbiota associations with common diseases and prescription medications in a population-based cohort. Nat Commun. 2018;9:2655. 10.1038/s41467-018-05184-7 29985401 PMC6037668

[48] Eckenberger J , Butler JC, Bernstein CN, Shanahan F, Claesson MJ. Interactions between medications and the gut microbiome in inflammatory bowel disease. Microorganisms. 2022;10:1963. 10.3390/microorganisms10101963 36296239 PMC9607329

[49] Cevallos SA , Lee J-Y, Tiffany CR, et al Increased epithelial oxygenation links colitis to an expansion of tumorigenic bacteria. mBio. 2019;10:10. 10.1128/mbio.02244-19 PMC6775460 31575772

[50] Nearing JT , Douglas GM, Hayes MG, et al Microbiome differential abundance methods produce different results across 38 datasets. Nat Commun. 2022;13:342. 10.1038/s41467-022-28034-z 35039521 PMC8763921

